# PROXIES OF FRAILTY AND INFLAMMATION PREDICT ALTERED WHITE MATTER MICROSTRUCTURE IN OLDER ADULTS

**DOI:** 10.1093/geroni/igac059.1156

**Published:** 2022-12-20

**Authors:** Andrew Bender, Sandra Duezel, Ulman Lindenberger, Ilja Demuth, Elisabeth Steinhagen-Thiessen, Thad Polk, Simone Kühn

**Affiliations:** Michigan State University, East Lansing, Michigan, United States; Max-Planck-Insitute for human developement, Berlin, Berlin, Germany; Max Planck Institute for Human Development, Berlin, Berlin, Germany; Charité Universitätsmedizin Berlin, Berlin, Berlin, Germany; Charité - Universitätsmedizin Berlin, Berlin, Berlin, Germany; University of Michigan, Ann Arbor, Michigan, United States; Max Planck Institute for Human Development, Berlin, Berlin, Germany

## Abstract

Age-related elevations in inflammation are associated with both neurodegeneration and increased frailty in older adults. Here we used state-of-the-art diffusion-MRI (dMRI) methods to examine how specific markers of white matter (WM), including fiber density [FD], fiber cross-section [FC], and extracellular cerebral spinal fluid [CSF] are linked to inflammation and frailty in a population-based aging cohort. We hypothesized that increased inflammation and frailty would be associated with reduced FD and FC and increased CSF. Participants included 255 older adults (Mage=70.46; SD=3.79 years; 42% women) recruited from the Berlin Aging Study-II (BASE-II) for MRI scanning. Measures of blood serum ferritin and grip strength (corrected for sex and body mass) served as proxies of inflammation and frailty, respectively. Processing of dMRI data (b=1000 s/mm2; 60-directions) followed the MRtrix3 fixel-based analysis (FBA) pipeline, extended for voxelwise estimation of FD, FC, and CSF, with data transformed to a sample-specific template. The voxelwise estimates of FD, FC, and CSF were then regressed on the measures of blood serum ferritin and grip strength, correcting for multiple comparisons. Greater inflammation (ferritin) and frailty (grip strength) predicted lower FD in the internal capsule and anterior commissure. Higher ferritin also predicted lower FC in dorsal cingulum and forceps minor. Finally, frailty, but not inflammation, predicted increased CSF in multiple regions. These findings demonstrate common and specific associations of inflammation and frailty with WM in older adults and highlight the utility of newer methods for characterizing WM alterations.

